# Infectious touching: Has COVID-19 changed our perceptions of social touch? A neural and behavioural study

**DOI:** 10.1093/scan/nsaf064

**Published:** 2025-06-26

**Authors:** Dana Zoabi, Elinor Abado, Simone Shamay-Tsoory, Leehe Peled-Avron

**Affiliations:** School of Psychological Sciences, University of Haifa, Abba Khoshey 199, Haifa, 3498838, Israel; The Integrated Brain and Behavior Research Center (IBBRC), University of Haifa, Abba Khoshey 199, Haifa, 3498838, Israel; School of Psychological Sciences, University of Haifa, Abba Khoshey 199, Haifa, 3498838, Israel; The Integrated Brain and Behavior Research Center (IBBRC), University of Haifa, Abba Khoshey 199, Haifa, 3498838, Israel; School of Psychological Sciences, University of Haifa, Abba Khoshey 199, Haifa, 3498838, Israel; The Integrated Brain and Behavior Research Center (IBBRC), University of Haifa, Abba Khoshey 199, Haifa, 3498838, Israel; Psychology Department, Bar-Ilan University, Ramat Gan, 5290002, Israel; Gonda multidisciplinary brain research center, Bar-Ilan University, Ramat Gan, 5290002, Israel

**Keywords:** COVID-19, Social touch, EEG, P1, LPP

## Abstract

Social touch serves as a pivotal element in stress reduction and cultivation of social bonds. The COVID-19 pandemic's constraints greatly affected social behaviour and may have reshaped human responses to such stimuli. We investigated the impact of COVID-19 on perceptions of interpersonal touch by comparing behavioural and electrophysiological data from pre- and peri-pandemic cohorts. Based on the vigilance-avoidance theory, we hypothesized that prolonged threat context of the pandemic would lead to reduced attentional and emotional engagement with social touch. Specifically, we expected that participants tested during the pandemic would rate social touch images as less pleasant and show lower amplitudes and longer latency in the P1 and lower amplitudes in the late positive potential (LPP) Electroencephalogram (EEG) components—markers of early attention and emotional processing—compared to pre-pandemic. Ninety participants rated the pleasantness of images showing human and inanimate touch or non-touch. As predicted, peri-pandemic participants rated social touch images as less pleasant than pre-pandemic participants. EEG analysis revealed a shift in P1 responses: while pre-pandemic participants showed higher P1 amplitudes for touch than non-touch stimuli, this distinction disappeared during the pandemic. No significant differences were found in LPP or P1. Results suggest that social distancing reduced the salience of interpersonal touch.

## Introduction

Social touch is defined as physical contact between human beings. It includes gestures such as hugging, holding hands, stroking or patting someone on the back, and can occur in various social interactions (Cascio et al. 2019). Touch is a fundamental human need and plays a crucial role in social interactions and communication. It can convey a wide range of emotions (McIntyre et al. 2022) and has been linked to physical and mental health benefits such as reduced stress and improved mood (Hertenstein et al. 2006, Hertenstein et al. 2009, Cascio et al. 2019) as well as enhanced sense of social connectedness to others (Eckstein et al. 2020). Given the magnitude of the effects of social touch, scholars have claimed that the mere observation of touch produces positive effects. For example, Schirmer et al. (2015) reported that observing social touch is associated with positive emotions and that characters in photos appear more positive and likeable when they touch each other than when they do not. Interestingly, people who suffer from mirror-touch synaesthesia find vicarious (observed) touch as pleasurable as actual touch (Pauli et al. 1997). In contrast, individuals with hereditary sensory and autonomic neuropathy, who have fewer tactile receptors involved in social touch (and cannot perceive social touch), find vicarious social touch less pleasurable (Olausson et al. 2002, Morrison et al. 2011). In line with the pleasant effects of observed touch, Peled-Avron et al. (2016) demonstrated that participants rate images of human social touch as evoking more pleasant emotions than images depicting non-social touch (between inanimate objects) or no touch, regardless of their empathy levels.

In an attempt to examine the neural processing of observed touch, scholars have studied event-related potentials (ERPs) (reviewed in Peled-Avron and Woolley 2022). ERPs are brain-generated electrical potentials that are associated with internal or external events (e.g. stimuli, responses, decisions; Bressler and Ding 2002). *P1* is an early ERP component that occurs approximately 110 ms after stimulus onset, is measured at parietal sites and reflects attention allocation (Wijers et al. 1997, Doesburg et al. 2008). During tasks involving stimulus discrimination, the amplitude of the P1 component increases when the participant concentrates on the stimulus. This heightened amplitude signifies a reduction in processing at unattended locations and minimized interference between attended and unattended information. Thus, the P1 component serves as a direct indicator of attentional resource allocation and effort, making it a neural marker for selective spatial attention (Heinze et al. 1994). P1 was found to have shorter latencies in response to social touch than to non-social touch stimuli, suggesting that participants allocate more attentional resources to social touch (Peled-Avron and Shamay-Tsoory 2017).

Another ERP component that researchers suggested to be associated with observed touch is the late positive potential (LPP) component (Peled-Avron and Shamay-Tsoory 2017). LPP has a positive amplitude that peaks 400–600 ms post-stimulus onset (Schupp et al. 2000, 2003, Peyk et al. 2009). LPP is typically measured at parietal electrodes and is higher in response to emotional stimuli than neutral stimuli (Schupp et al. 2000, Hajcak et al. 2010). Unlike the P1 component, LPP is associated with the processing of higher and more complex cognitive and emotional information, such as affective images (Olofsson et al. 2008), human voices (Schirmer and Gunter 2017), facial expressions (Hartigan and Richards 2017) and felt touch (Ackerley et al. 2013). LPP was shown to reflect vigilance towards emotional stimuli (Kausche et al. 2022). For example, increased LPP amplitudes have been demonstrated in response to observed touch (Peled-Avron and Shamay-Tsoory 2017, Adler and Gillmeister 2019, Schirmer and McGlone 2019), possibly reflecting enhanced social-emotional processing in response to observed touch.

Previous studies demonstrated that individuals with higher autistic traits have a strong aversion to interpersonal touch which was associated with higher LPP amplitudes and with shorter P1 latencies. These serve as a marker of anxiety bias, and as a marker for detection and allocation of attention to social touch (Peled-Avron and Shamay-Tsoory 2017). These findings suggest that the early ERP component (P1) reflects enhanced allocation of attentional resources to stimuli, whereas the later ERP components (LPP) reflect emotional and social reactions to observed touch (Peled-Avron and Woolley 2022).

Human perception of social touch is influenced by a variety of social factors that extend beyond its physical characteristics. Context plays a key role, as (Sailer et al. 2024) highlight that the features of touch—such as its type and location where it is administered—are less pronounced than factors like need fulfilment (particularly relatedness) and the perceived intentions behind the touch. These findings emphasize the importance of understanding touch within its broader social and psychological context. Individual differences also shape the perception of touch. For instance, sensory processing sensitivity and empathic ability influence how people experience tactile interactions (Peled-Avron & Woolley, 2022). (Silvestri et al. 2024) found that affective touch reduces arousal and the perceived unpleasantness of emotional stimuli, though these effects vary across individuals. Broader social influences, such as the COVID-19 pandemic, have also had profound effects on touch perception. Von Mohr et al. (2021) observed that shifting norms and increased physical distancing during the pandemic altered the emotional salience of touch, underscoring its role in mitigating loneliness. Moreover, (Gallace and Spence 2010) demonstrated that touch can influence social behaviours, such as compliance, bonding, and attitude formation, even when it is not consciously remembered. Collectively, these findings underscore the complex interplay between contextual factors, individual differences, and social influences in shaping the emotional and social significance of touch.

COVID-19 is a severe respiratory disease that first appeared in 2019 and spread rapidly across the globe. It caused thousands of deaths, thus prompting the World Health Organization to declare it as a pandemic in March 2020. The COVID-19 pandemic brought about significant changes in social behaviour as people had to adapt to new norms of social restrictions and keep their distance from one another to prevent the spread of the disease (Abbasi 2020). All ordinary social behaviours, such as meeting with friends and family members, hugging and shaking hands suddenly became unacceptable and less prevalent (Bavel et al. 2020). Restrictions on these behaviours increased levels of social isolation and loneliness (Horigian et al. 2021). Quarantine measures, physical separation and the closure of public spaces all reduced opportunities for social interaction and support. Indeed, touch—one of the factors shown to diminish loneliness and increase social connectedness (Heatley Tejada et al. 2020)—was less available during the pandemic.

In the current study, we sought to examine whether the pandemic has influenced people’s perceptions of social touch. Hence, the aim of the present study was to investigate behavioural and neural changes in perception of interpersonal touch brought about by COVID-19. What we know about perceived interpersonal touch during COVID-19 is limited. The assumption was that the pandemic would have an impact on people's attitudes towards social touch and perceived social touch. Yet the question remains as to whether social restrictions may have increased or decreased the pleasantness associated with social touch. While behavioural reactions to social touch were studied to some extent during and after the pandemic, the neural underpinnings of this behaviour have not been studied. Therefore, this study aims to characterize the behavioural *and* neural changes in perception of interpersonal touch brought on by the COVID-19 pandemic, by comparing data collected before the pandemic [for behavioural data see Peled-Avron et al. (2016), and for neural data see Peled-Avron and Shamay-Tsoory (2017)] to data collected during the pandemic. This comparison can advance our knowledge regarding the effects of COVID-19 on perceived social touch, to enable us to better understand the impact of the pandemic on people's social well-being and inform efforts to mitigate the negative effects of isolation and loneliness.

In view of the reduction in social touch during the pandemic and the emotional toll of prolonged isolation, we hypothesized that there would be differences between pre- and peri-COVID-19 responses to observed touch. Specifically, we drew on the vigilance-avoidance theory (Mogg et al. 2004, Derakshan et al. 2007), which proposes a two-phase attentional response to threat: initial hypervigilance, followed by attentional avoidance to reduce emotional distress. While the theory was originally applied to short-term threat processing, we apply it here *as a conceptual framework* to a prolonged social context. We hypothesized that as the pandemic progressed, attentional avoidance mechanisms would dominate, leading to reduced vigilance towards social touch. Our peri-COVID-19 measurement occurred between Israel’s second and third lockdowns, a period that we interpret as aligning with the avoidance phase of the vigilance-avoidance model. Therefore, we hypothesized that peri-COVID-19 participants would rate social touch photos as less pleasant than in pre-COVID-19 studies (Peled-Avron et al. 2016). Additionally, we expected lower LPP and P1 amplitudes, reflecting reduced attentional and emotional engagement with social touch. Finally, we hypothesized that P1 latency would be longer in the peri-COVID-19 group, suggesting slower initial processing of social touch, consistent with reduced orientation towards social stimuli.

## Materials and methods

### Participants

The pre-COVID-19 group originally included 54 participants (aged 18–39, *M* = 23.03, *SD* = 4.09), as reported in Peled-Avron et al. (2016). However, two participants were excluded due to missing data, resulting in a final sample of 52 participants in this group.

The peri-COVID-19 group included 43 participants (25 female), recruited via a university platform or a Facebook ad. Participants were asked: “Which of the following best describes your gender? (check one): Woman; Man; Nonbinary, genderfluid, or gender non-conforming; Prefer not to answer.” All selected either “woman” or “man.” Participants received course credit or payment. Their ages ranged from 18 to 42 (*M* = 26.15, *SD* = 5.81). Five participants were excluded from the electroencephalogram (EEG) analyses due to excessive noise (i.e. >25% trial rejection; Lopez-Calderon et al. 2014), leaving a final sample of 38 participants.

Sample size was determined using G*Power (version 3.1.9.7; Faul et al. 2009) to detect a medium effect size (partial eta^2^ = 0.06) with power = 0.80, *α* = 0.05, in a 2 (Touch) × 2 (Object) × 2 (Group) design. A minimum of 31 participants per group was required, which was met in both groups.

Data collection took place from 1 August 2021 to 28 April 2022. All participants were right-handed and reported normal or corrected-to-normal vision. A screening interview confirmed that all participants had no history of psychiatric or neurological disorders. Participants gave signed informed consent to participate in the study. Our recruitment strategy aimed to closely align participant characteristics with those of the pre-COVID cohort to ensure consistency and comparability across study phases. We verified post hoc that the recruited cohort closely matched the pre-COVID cohort in key demographic variables. This verification was conducted to ensure comparability and to reduce potential biases stemming from recruitment differences.

This study was approved by the Ethics Review Committee of the University of Haifa (no. 231/21).

### Stimuli task and design

**Figure 1.**
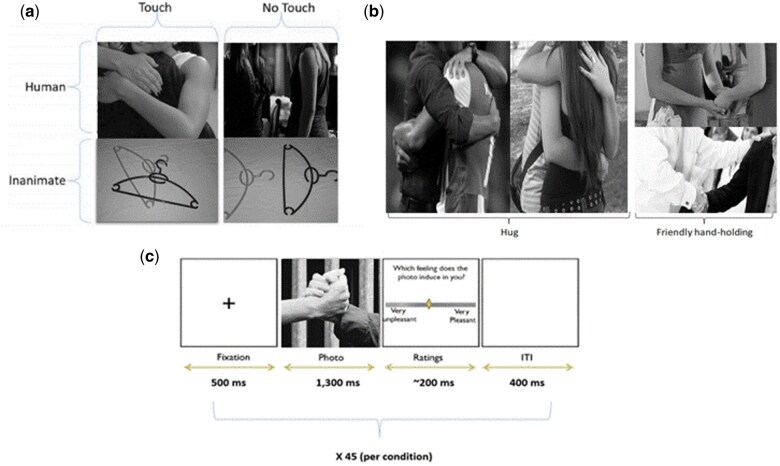
Examples of the task stimuli and design. (a) Sample stimuli images depicting a natural social interaction in a public setting between two women that did or did not involve touch and control images depicting two inanimate objects either touching or not. (b) Sample images depicting social interactions involving touch between two males and two females. (c) Trial scheme in the computerized paradigm: A fixation cross followed by the photo and rating screen, with an interstimulus interval between each trial. Each condition contains 45 photos counterbalanced between four blocks. ITI = intertrial interval.

The computerized task used in this experiment is identical to the one described by Peled-Avron et al. (2016). Specifically, a total of 45 photos were shown, divided into four conditions—humans or objects either touching or not touching—forming a 2 × 2 design of object (human, inanimate) × touch (with, without). The human touch images depicted a hug, a handshake, a tap on the shoulder or friendly handholding (Fig. 1a). The inanimate contact pictures depicted two common objects without any commercial logos (e.g. cutlery, clothing items) touching one another. In the non-touch condition, humans or objects were shown without touching. This combination of touch and object type yielded a 2 × 2 factorial design. Previous studies using the same paradigm found that human touch photos were rated as significantly more pleasant than other categories (human non-touch, object touch and object non-touch; Peled-Avron et al. 2016, Peled-Avron and Shamay-Tsoory 2017).

The task was administered to participants who were seated about 60 cm from a 21 CRT monitor. E-Prime 2.2 Psychological Software was used for stimulus presentation and experimental control. To account for any potential low-level visual changes across the stimulus categories, participants were shown monochromatic images that were 15 cm × 10 cm in size and had fixed luminance (Johannes et al. 1995). During the experiment, participants were instructed to rate the extent of pleasantness or unpleasantness evoked by each photo. We employed a bipolar valence visual analog scale (VAS) to quantify participants’ subjective responses. Subsequently, the VAS ratings were converted into numerical values offline, ranging from 0 (representing unpleasant feelings) to 100 (representing pleasant feelings). In accordance with the Attitudes to Problem-Solving Ability (IAPS) protocol (Lang et al. 1998), participants were instructed as follows: “At one extreme of the scale, you feel completely unpleasant, unhappy, annoyed, dissatisfied, melancholic or despairing. On the other extreme, you experience complete satisfaction, joy, happiness, contentment, or hope.” Further, participants were informed that a score at the scale's middle point represented a completely neutral state that was neither pleasant/happy nor unpleasant/sad.

As described in Peled-Avron et al. (2016), male participants were shown images depicting social touching between men, whereas female participants were shown images depicting social touching between women (see Fig. 1b). Moreover, all the portrayed social interactions took place between same-sex friends. This methodological choice was made to reduce variability and ensure greater homogeneity in the emotional and attentional responses to the stimuli. Furthermore, since the new sample was compared to the previous sample, we constructed the experiment to be the same (Peled-Avron et al. 2016). Research suggests that individuals may respond differently to social touch images based on factors such as gender (Suvilehto et al. 2023). By matching participant gender to the gender shown in the images, we aimed to standardize the stimuli, thereby minimizing potential sources of variability that could arise from cross-gender differences in touch perception. This approach allowed us to assess more reliably the neural and behavioural responses to social touch while focusing on the main variables of interest. The human condition photographs depicted the neck and down with no faces shown in order to keep the stimuli simple and reduce any potential confounding effects. The stimuli were presented in four blocks of 45 trials each, for a total of 180 trials. Every trial started with a fixation cross shown for 500 ms, followed by a picture for 1300 ms, and an inter-trial period of a blank screen for 400 ms. Each participant was shown a different set of photos in each of the four blocks, in random order (see Fig. 1c).

### EEG data acquisition

During the behavioural task, EEG was recorded from 32 scalp sites using active, gel-based Ag/AgCl electrodes mounted in an elastic cap (g.Nautilus, g.tec medical engineering GmbH) using an extended 10–20 system. The EEG signals were digitally amplified and sampled at 500 Hz. Data were wirelessly transmitted using 2.4 GHz band. Impedances were maintained below 5 kΩ.

### Behaviour analysis

To test participants’ emotional response to the four conditions, we conducted a mixed analysis of variance (ANOVA), with *group* as the between-subjects factor (pre-COVID-19 and peri-COVID-19) and *touch* (touch, no touch) and *object* (human, inanimate) as within-subject factors. The emotional ratings of the photos served as the dependent variable. Behavioural data were analysed using IBM SPSS Statistics (version 27.0.1). The degrees of freedom were corrected using Greenhouse–Geisser epsilon values and Bonferroni correction for multiple comparison.

### EEG data processing

Data were analysed using EEGLab (version 2021.0; Delorme and Makeig 2004) and ERPLab plugin (Lopez-Calderon et al. 2014) running on MATLAB (MathWorks; version R2021b) routines. Raw EEG data were re-referenced offline to the digital average of the 32 EEG electrodes. EEG deflections resulting from eye blinks were corrected using independent component analysis (ICA). Any remaining artefacts that exceeded ±100 µV in amplitude were rejected.

### Event-related potentials analysis

Event-related potentials were determined by averaging the 1300 ms segmented trials separately in each stimulus condition (human touch, human non-touch, object touch, and object non-touch). The average waveforms were smoothed by applying a 30 Hz low-pass filter and 0.01 Hz high-pass filter, and were baseline corrected to the signal recorded 200 ms before stimulus onset. Based on LPP literature discussing visual processing of the affective content in photos (Schupp et al. 2000, 2003, Peyk et al. 2009), LPPs were analysed for the vertex electrodes (Pz, Cz, and Fz; Schupp et al. 2003). LPP amplitudes were scored as the mean amplitude in the time interval from 400 to 600 ms following stimulus onset. The statistical analysis of the P1 component was limited to the parietal sites P7 and P8, which are commonly used for P1 analyses (Wijers et al. 1997, Doesburg et al. 2008). The peak of the P1 component was defined for each participant as the largest positive peak amplitude between 100 and 150 ms. Visual inspection ensured that these values represented actual peaks rather than epoch end-points. LPP, P1 amplitudes and P1 latency were compared before and after COVID-19. Interactions with type of photo were assessed using mixed ANOVA, with *group* as the between-subjects factor (pre-COVID-19 and peri-COVID-19) and *touch* (touch, no touch) and *object* (human, inanimate) as within-subject factors. The dependent variable was the LPP amplitude, P1 amplitude or P1 latency.

## Results

### Behaviour

**Figure 2.**
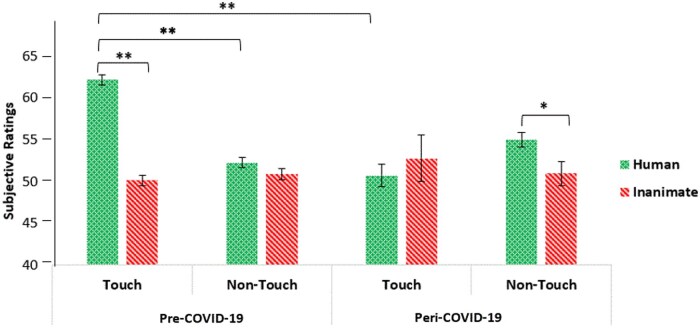
Subjective behavioural ratings for the pre- and peri-COVID-19 groups. Participants in the peri-COVID-19 group rated human touch photos as inducing less pleasant emotions than did participants in the pre-COVID-19 group. In the pre-COVID-19 group, subjective ratings of human touch photos were higher than ratings both of inanimate touch photos and of non-touch photos. In the peri-COVID-19 group, however, participants rated human non-touch as more pleasant than inanimate non-touch. Main effects were found for the touch condition and the object condition. Error bars represent standard error of the mean. *P < .05, **P < .001.

To examine the hypothesis that the perception of social touch was affected by COVID-19, the analysis revealed a significant three-way interaction between touch, object, and COVID-19 (*F*(1, 106) = 33.399, *P* < .001, partial η^2^ = .24; see Fig. 2).

To understand the three-way interaction, we conducted separate analyses for the pre- and peri-COVID-19 groups.

In the pre-COVID-19 group, there was a significant interaction between touch and object (*F*(1, 64) = 77.91, *P* < .001, partial η^2^ = .549). Participants rated photos depicting human touch (*M* = 62.19, *SD* = 5.01) as more pleasant than photos depicting inanimate touch (*M* = 50.14, *SD* = 5.71, *P* < .001), while no significant differences were found in the no-touch condition (*M* = 52.26, *SD* = 5.20 for human; *M* = 50.88, *SD* = 5.20 for inanimate*, P* = .205). Additionally, there were main effects of touch (*F*(1, 64) = 46.827, *P* < .001, partial η^2^ = .423) and object (*F*(1, 64) = 99.048, *P* < .001, partial η^2^ = .607), indicating that participants rated touch photos as more pleasant (*M* = 56.17, *SD* = 5.37) than no-touch photos (*M* = 51.57, *SD* = 5.33) and human photos as more pleasant than inanimate photos.

In the peri-COVID-19 group, the interaction between touch and object was marginally significant (*F*(1, 42) = 4.004, *P* = .052, partial η^2^ = .087). In the touch condition, no differences were found between photos depicting human touch (*M* = 50.71, *SD* = 18.42) and inanimate touch (*M* = 52.78, *SD* = 9.23, *P* = .466). However, in the no-touch condition, human photos (*M* = 55.04, *SD* = 8.73) were rated as more pleasant than inanimate photos (*M* = 50.93, *SD* = 5.80, *P* = .012).

These findings indicate that while the pre-COVID-19 group showed a clear preference for human touch over inanimate touch, this distinction diminished in the peri-COVID-19 group. This suggests that the perception of social touch was significantly altered during the pandemic, consistent with our hypothesis.

Additionally, we performed a direct comparison between the pre-COVID-19 and peri-COVID-19 groups for each condition (applying Bonferroni correction) to focus on the differences *between the two groups* in each condition. Follow-up *t*-tests showed that there was a significant difference between pre-COVID-19 and peri-COVID-19 ratings in the human touch condition (*t*(106) = 4.775, *P* < .001) such that pre-COVID-19, participants rated human touch photos as more positive (*M* = 62.187, *SD* = 5.012) than peri-COVID-19 (*M* = 50.706, *SD* = 18.42). In addition, there was a significant difference between pre-COVID-19 and peri-COVID-19 ratings in the non-touch human condition (*t*(106) = −2.069, *P = *.041), such that peri-COVID-19, participants rated non-human touch photos as more positive (*M* = 55.038, *SD* = 8.73) compared to pre-COVID-19 (*M* = 52.264, *SD* = 5.19). No other significant differences were found for inanimate conditions.

### P1 amplitude

**Figure 3.**
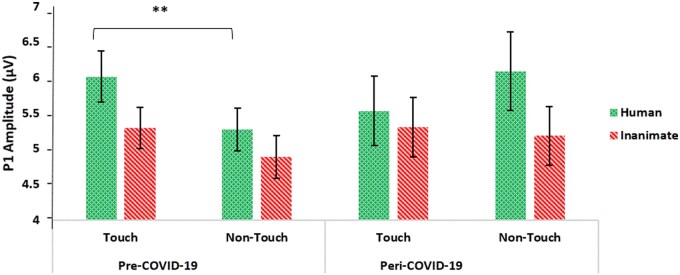
P1 amplitudes for the pre- and peri-COVID-19 groups (averaged between P7 and P8 electrodes, 100–150 ms post-stimulus). In the pre-COVID-19 group, the P1 amplitudes showed a more positive response when viewing photos with touch compared to photos without touch. Yet after the pandemic no significant differences emerged between touch photos and non-touch photos. Error bars represent standard error of the mean **P < .001.

**Figure 4.**
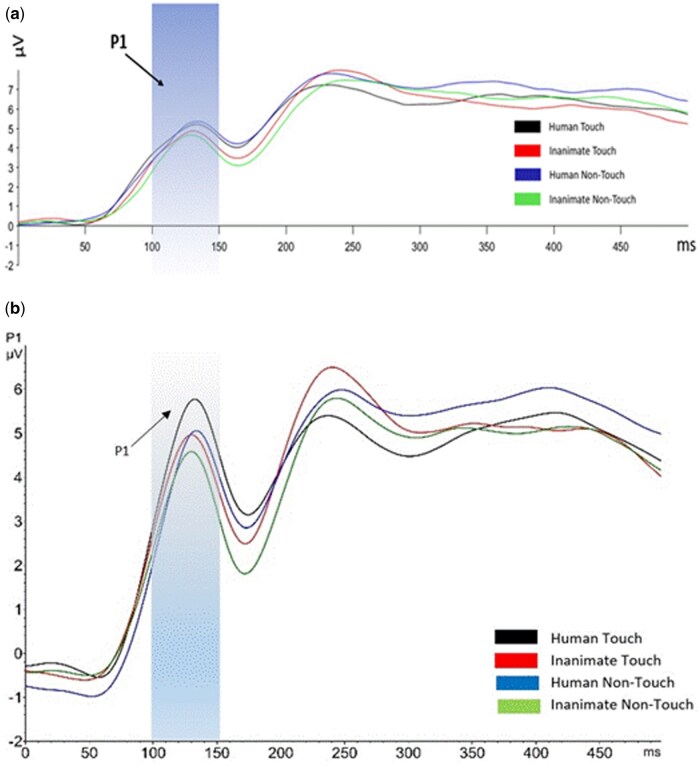
Onset-synchronized grand-average ERP P1 component measured under the indicated experimental conditions. The P1 component was averaged between P7 and P8 electrodes. The shaded areas represent the time windows for P1 (approximately 130 ms post-stimulus). (a) P1 amplitudes for the peri-COVID-19 group; (b) P1 amplitudes for the pre-COVID-19 group (adapted from Peled-Avron et al. 2016).

To test whether P1 amplitudes were affected by the pandemic, we conducted a mixed ANOVA analysis, with touch (touch, no-touch) and object (human, inanimate) as within-subject factors and COVID-19 (pre-COVID-19, peri-COVID-19) as a between-subjects factor. The analysis revealed a significant three-way interaction between touch, object, and COVID-19 (*F*(1, 90) = 5.972, *P* = .016, partial η^2^ = .062; see Figs. 3 and 4). A significant interaction emerged between touch and COVID-19 (*F*(1, 90) = 20.85, *P* < .001, partial η^2^ = .188). Specifically, in the pre-COVID-19 group, P1 amplitudes were more positive when participants viewed touch photos compared to non-touch photos (*F*(1, 53) = 26.238, *P* < .001, partial η^2^ = .226). In contrast, in the peri-COVID-19 group, no differences were found between touch and non-touch photos (*F*(1, 37) = 2.766, *P* = .1, partial η^2^ = .03). This aligns with our hypothesis that P1 amplitudes will be lower in the peri-COVID-19 compared to pre-COVID-19.

We further examined the COVID-19 conditions separately.

In the pre-COVID-19 condition, no interaction was found between touch and object (*F*(1, 53) = 2.24, *P* = .14, partial η^2^ = .041). However, significant main effects emerged for both touch (*F*(1, 53) = 42.423, *P* < .001, partial η^2^ = .445) and object (*F*(1, 53) = 20.866, *P* < .001, partial η^2^ = .282). P1 amplitudes were more positive when participants viewed photos depicting touch (*M* = −1.444, *SD* = 0.744 for human touch; *M* = −0.489, *SD* = 0.382 for inanimate touch) compared to photos with no touch (*M* = −0.722, *SD* = 0.429 for human no-touch; *M* = −0.624, *SD* = 0.442 for inanimate no-touch). Additionally, human photos elicited higher P1 amplitudes than inanimate object photos.

**Figure 5.**
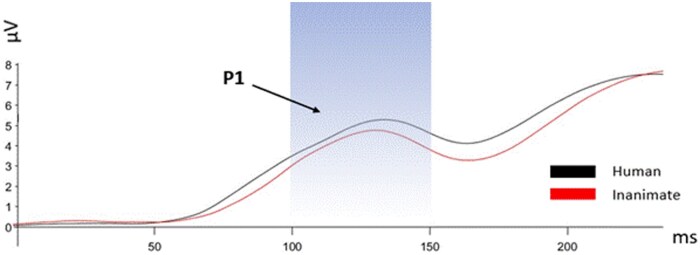
Onset-synchronized grand-average ERP P1 waveforms peri-COVID-19 for the human (black) and inanimate (red) conditions measured at electrodes P7 and P8. The blue shaded area indicates the time window for P1 (approximately 130 ms post-stimulus). The P1 amplitude was significantly lower for the inanimate objects condition (*P* < .05).

In the peri-COVID-19 condition, no interaction was found between touch and object (*F*(1, 37) = 3.236, *P* = .08, partial η^2^ = .08). As shown in Fig. 5, a main effect emerged for object (*F*(1, 37) = 8.106, *P* = .007, partial η^2^ = .18), indicating that P1 amplitudes were more positive when participants viewed human photos than when they viewed inanimate object photos. Specifically, P1 amplitudes for human touch photos (*M* = −0.659, *SD* = 1.010) and inanimate touch photos (*M* = −0.476, *SD* = 0.746) were more positive than for no-touch human (*M* = −0.824, *SD* = 0.989) and inanimate no-touch photos (*M* = −0.619, *SD* = 0.874).

A significant interaction emerged between touch and COVID-19 (*F*(1, 90) = 20.85, *P* < .001, partial η^2^ = .188). Specifically, in the pre-COVID-19 group, P1 amplitudes were more positive when participants viewed touch photos compared to non-touch photos (*F*(1, 53) = 26.238, *P* < .001, partial η^2^ = .226). In contrast, in the peri-COVID-19 group, no differences were found between touch and non-touch photos (*F*(1, 37) = 2.766, *P* = .1, partial η^2^ = .03).

Additionally, we performed a direct comparison between the pre-COVID-19 and peri-COVID-19 groups for each condition for P1 amplitude (applying Bonferroni correction) to focus on the differences *between the two groups*. Follow-up *t*-tests showed there were no significant differences between the groups in all four conditions (all *p’*s > 0.166).

### P1 latency

We conducted a mixed ANOVA analysis to test whether P1 latency before the pandemic was shorter than during the pandemic, with *touch* (touch, no-touch) and *object* (human, inanimate) as within-subject factors and *COVID-19* (pre-COVID-19, peri-COVID-19) as a between-subjects factor. No three-way interaction was found between touch, object, and COVID-19 (*F*(1, 90) = 1.458, *P* = .23, partial *η^2^* = .016). No other interactions or main effects were found (*P* >.05), contrary to our hypothesis.

### Late positive potential

**Figure 6.**
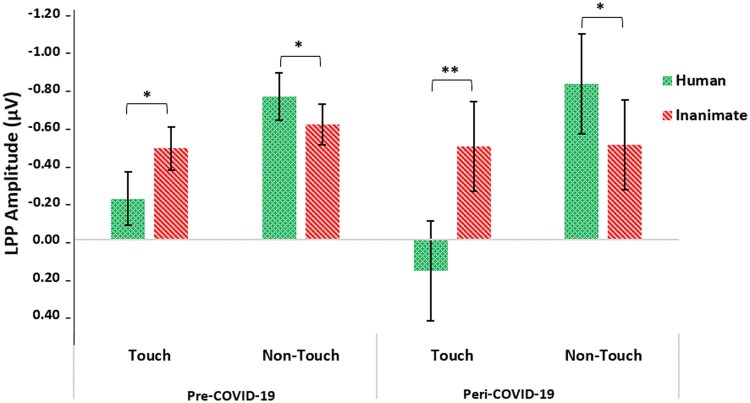
LPP average amplitudes for the pre- and peri-COVID-19 groups (averaged between Fz, Cz, and Pz electrodes, 400–600 ms post-stimulus). In both the pre- and peri-COVID-19 groups, the LPP amplitudes were more positive during observation of human touch. The LPP amplitudes were also more positive when participants observed non-touch human photos than when they observed non-touch inanimate photos. A main effect was found for the touch condition. Error bars represent standard error of the mean *P < .05 **P < .001.

**Figure 7.**
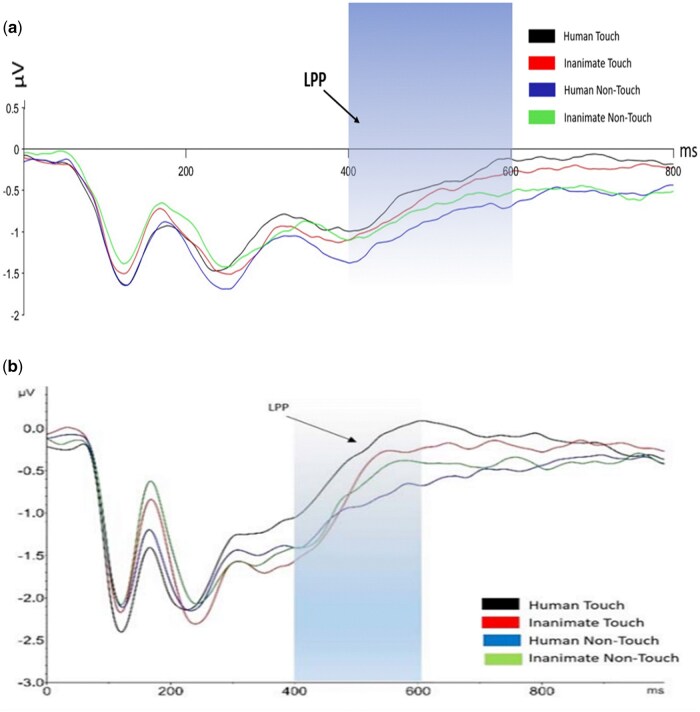
Onset-synchronized grand-average ERP LPP component measured under the indicated experimental conditions. The LPP component was averaged between the midline electrodes Fz, Cz, and Pz. The shaded areas indicate the time windows for LPP (400–600 ms post-stimulus). (a) LPP amplitudes for the peri-COVID-19 group. (b) LPP amplitudes for the pre-COVID-19 group (adapted from Peled-Avron et al. 2016).

To examine the hypothesis that LPP amplitude will be lower in the peri-COVID-19 group compared to the pre-COVID-19 group, we conducted a mixed ANOVA analysis, with touch (touch, no-touch) and object (human, inanimate) as within-subject factors and COVID-19 (pre-COVID-19, peri-COVID-19) as a between-subjects factor. LPP analysis revealed a significant three-way interaction between touch, object, and COVID-19 (*F*(1, 90) = 8.87, *P* = .004, partial η^2^ = .09; see Figs. 6 and 7).

COVID-19 groups were then examined separately to explore the source of this interaction.

In the pre-COVID-19 group, a significant interaction emerged between touch and object (*F*(1, 53) = 18.856, *P* < .001, partial η^2^ = .262). In the touch condition, higher LPP amplitudes were found when participants observed human touch photos (*M* = −0.2185, *SD* = 0.143) compared to inanimate touch photos (*M* = −0.4854, *SD* = 0.114, *P* = .013). Conversely, in the non-touch condition, the LPP amplitudes were more positive towards inanimate photos (*M* = −0.6117, *SD* = 0.107) than when participants observed human photos (*M* = −0.7607, *SD* = 0.126, *P* = .05).

In the peri-COVID-19 group, a significant interaction also emerged between touch and object (*F*(1, 37) = 29.29, *P* < .001, partial η^2^ = .442). Similarly, in the touch condition, higher LPP amplitudes were found when participants observed human touch photos (*M* = 0.1651, *SD* = 0.266) compared to inanimate touch photos (*M* = −0.4953, *SD* = 0.238, *P* < .001). Conversely, in the non-touch condition, the LPP amplitudes were more positive towards inanimate photos (*M* = −0.5060, *SD* = 0.239) than when participants observed human photos (*M* = −0.8279, *SD* = 0.267, *P* = .039).

Additionally, we performed a direct comparison between the pre-COVID-19 and peri-COVID-19 groups for each condition for LPP amplitude (applying Bonferroni correction), to focus on the differences *between the two groups*. Follow-up *t*-tests showed there were no significant differences between the groups in any of the four conditions (all *p’*s > 0.175). Overall, contrary to our hypothesis, no significant differences in LPP amplitudes were found between the peri-COVID-19 sample and the pre-COVID-19 sample.

## Discussion

In the current study, we examined how perceptions of interpersonal touch in social interactions were affected by the COVID-19 pandemic. Specifically, we compared pre- and peri-COVID-19 behavioural and neural responses to observed social touch. The stimuli described in Peled-Avron et al. (2016)  and Peled-Avron and Shamay-Tsoory (2017) enabled us to compare behavioural and neural data (Peled-Avron et al. 2016, Peled-Avron and Shamay-Tsoory 2017, respectively) collected prior to COVID-19, to a new set of data collected peri-COVID-19. We focused on the P1 and LPP ERP components, which reflect early and late attentional allocation and emotional processing, respectively. We hypothesized that during the pandemic, individuals may exhibit diminished responses to social touch, suggesting reduced attentional allocation or sensitivity to social touch stimuli. As predicted, we found behavioural changes in social touch perception: participants in the peri-COVID-19 group rated photos with *touch* as less pleasant than did participants in the pre-COVID-19 group. Moreover, participants in the peri-COVID-19 group rated photos with *humans* as less pleasant than did participants in the pre-COVID-19 group. It is interesting to note that the peri-COVID-19 group exhibited no differences in pleasantness ratings between human touch and inanimate touch photos, whereas for the non-touch conditions, participants perceived photos of non-touching humans as more pleasant than photos of non-touching inanimate objects. This pattern was not found before COVID-19. The results of the current study suggest that the pandemic may have had an impact not only on behavioural responses, but it also affected neural processing of touch stimuli, resulting in different patterns in P1 amplitudes. In the pre-COVID-19 group, the P1 amplitudes were higher when participants observed touch compared to non-touch photos, whereas in the peri-COVID-19 group, P1 amplitudes did not differ between touch and non-touch photos. No changes were found in LPP amplitudes or in P1 latencies for the peri-COVID-19 sample compared to the pre-COVID-19 sample.

The decreased pleasantness ratings for photos depicting touch and humans in the peri-COVID-19 group may have been influenced by various factors associated with the pandemic. First, concerns about potential transmission of the virus through touch as well as increased awareness of hygiene practices may have led to the reduced pleasantness rating for touch-related stimuli. The changes in participants’ perceptions and evaluations of social touch and human stimuli after COVID-19 may represent a defensive response serving to mitigate potential risks and threats associated with the pandemic. By devaluing the pleasantness of touch and perceiving photos with human touch as less pleasant, individuals may be adopting a protective mechanism to avoid potential harm and reduce their susceptibility to the virus (Jones 2007, Oaten et al. 2009). Moreover, these results may be aligned with the finding that perceived vulnerability to disease predicts conformist attitudes (Murray and Schaller 2012). When collectively facing the threat of COVID-19, individuals may have adopted more cautious behaviours, conformed to public health guidelines and sought to minimize the risk of infection. Consequently, avoiding physical contact, including social touch, may be seen as a conformist response to the prevailing norms and expectations surrounding personal safety and health.

The results of the current study suggest that the pandemic may have influenced behavioural responses and potentially neural processing of touch stimuli, as reflected in different patterns of P1 amplitudes. Specifically, prior to COVID-19, stimuli depicting human touch elicited a stronger neural response than stimuli depicting humans who are not touching or stimuli depicting inanimate objects either touching or not touching. In contrast, during the pandemic the differentiation between social touch and social non-touch diminished. Although overall P1 amplitudes did not differ significantly between groups, the pattern of response to different touch conditions changed, indicating possible modulation of early attention processes. These findings may reflect shifts in attention or changes in the salience of touch-related information due to the altered circumstances brought about by the pandemic. Moreover, these findings may be related to attentional bias. Attentional bias refers to the tendency to selectively attend to certain stimuli over others (Abado et al. 2020). In the context of touch perception and social stimuli, attentional biases can influence how individuals allocate their attention to these stimuli, subsequently affecting the P1 component (Clark and Hillyard 1996). Prior to COVID-19, participants may have shown a bias in attention towards touch, as evidenced by the higher P1 amplitudes for photos with touch compared to photos without touch. This may suggest that touch­related stimuli captured attention more effectively. Additionally, participants exhibited a bias towards photos depicting humans over those depicting inanimate objects, indicating a preference for attending to social stimuli. During the pandemic, however, the interaction between touch and object was not significant, indicating a reduction in the attentional bias towards touch, which was found before the pandemic. Taken together, while direct COVID-19 group differences in P1 amplitudes were not observed, the altered pattern of responses suggests that attentional bias for social touch, reflected in early processing of visual stimuli, may have been modulated by the pandemic and its associated changes in social interactions.

Moreover, Krusemark and Li (2013) showed that perception of disgusting pictures is associated with reduced P1 amplitudes, whereas neutral or positive affect pictures elicit larger P1 amplitudes. This finding suggests that the P1 component is sensitive to emotional states. In the context of our study, we can speculate that the observed decrease in P1 amplitudes when participants observed social touch photos may be attributed to feelings of disgust. The implication is that P1 amplitudes were less positive during the pandemic than before the pandemic, possibly due to feelings of disgust when viewing the photos. This is noteworthy considering that disease prevention has been associated with the emotion of disgust (Jones 2007, Oaten et al. 2009). To further enhance our understanding of the relationship between P1 amplitudes and perceived disgust in our study, it would have been beneficial to include a subjective rating task in which participants were asked to rate the photos on a scale specifically designed to measure the degree of disgust they experienced.

The LPP component, which reflects later-stage attentional allocation and emotional processing, may capture varying types of engagement depending on the context. In line with prior research, higher LPP amplitudes have been associated with vigilance towards emotional stimuli (Schupp et al. 2000). However, the LPP is not exclusively indicative of negative processing; it may also reflect heightened attentional and emotional engagement with positively valanced stimuli under certain conditions such as social touch (Schirmer and McGlone 2019). For example, pre-pandemic responses may have reflected positive engagement with social touch, as evidenced by higher LPP amplitudes in response to human touch photos. Conversely, during the pandemic, the heightened vigilance induced by infection risk and altered social norms may have shifted the interpretation of LPP amplitudes to reflect more negative engagement with the same stimuli.

In the wake of the COVID-19 pandemic, it is important to examine the implications and impact of its social restrictions on human social functioning. Social touch is a crucial part of everyday social interactions. Whereas other researchers have examined perceptions of social touch during and after COVID-19, to our knowledge this study is the first to compare perceptions of social touch to a set of data collected prior to the pandemic, while also examining the neural mechanisms underlying these perceptions. The literature regarding the effects of COVID-19 on social touch is inconsistent. Some researchers claim that social restrictions may increase the craving for social touch in the same way that fasting increases the craving for food (e.g. Von Mohr et al. 2021, Ujitoko et al. 2022). Others argue that the social restrictions dictated by COVID-19 have changed how humans perceive touch, such that touch is now perceived as a threat signal that may even generalize into reduced pleasant reactions to observed touch (Massaccesi et al. 2021). Our results support the second argument and are in line with a study by Massaccesi et al. (2021), which demonstrated that images depicting crowds and large gatherings were rated as less pleasant during the pandemic period compared to the pre-pandemic period. Moreover, participants who reported overall greater physical isolation, stronger feelings of social closeness and higher perceived threat of COVID-19, gave more positive ratings to images depicting individuals alone and in very small groups. Our findings are also in line with Kühne et al. (2022), who found that following the pandemic, participants perceived situations in which two individuals were depicted wearing face masks and not interacting with one another as more positive. One important and surprising insight emerging from the current study is that COVID-19 has had an impact on human perception of social touch, such that observing non-social touch evoked more positive emotions than observing human touch.

Numerous studies have demonstrated that both observed touch and experienced touch activate similar brain regions (e.g. Keysers et al. 2004, Ebisch et al. 2008), suggesting that witnessing someone else being touched and experiencing touch oneself engage the same neural mechanisms (Schirmer et al. 2015). Nevertheless, it is crucial to recognize that the actual physical experience may differ, as observing photos may not fully capture the complex and dynamic nature of tactile interactions (Abraira and Ginty 2013), especially those that occurred during the pandemic. The absence of physical and sensory experience in our study may limit the generalizability of our findings. Real-life encounters involving touch can evoke heightened feelings of threat or intimacy, potentially exerting an influence on individuals’ perceptions and responses that differs from the effect of static visual representations (Saarinen et al. 2021). Therefore, future research should consider incorporating real-life touch scenarios to gain a more comprehensive understanding and provide deeper insights into the complex interplay between social touch perception and COVID-19.

A limitation of the current study is that we did not collect information on participants’ individual differences in their experiences and attitudes towards touch, such as through validated measures, e.g. the Touch Experiences and Attitudes Questionnaire (TEAQ) (Trotter et al. 2018). Individual differences in touch experiences and preferences could influence how participants perceive and evaluate touch stimuli, potentially contributing to variability in the results. Future research should consider incorporating such measures, to better account for these individual differences and provide a more nuanced understanding of how personal attitudes towards touch interact with situational factors, such as the impact of the COVID-19 pandemic, on touch perception.

A potential limitation of our study lies in the breadth of the pleasantness-unpleasantness rating scale used to assess participants’ responses. While the scale was designed to capture a general sense of emotional valence, including satisfaction, dissatisfaction, and sadness, it is possible that participants interpreted and rated sadness associated with a perceived loss of touch in daily life during peri-COVID-19. Studies have shown that the social distancing regulations during the COVID-19 pandemic significantly reduced opportunities for interpersonal touch, leading to feelings of longing for touch which increased with the duration and severity of the regulations (Meijer et al. 2022, Hasenack et al. 2023). Touch deprivation was also associated with negative mood states and depression (Field et al. 2020). In our study, the wide-ranging nature of the rating scale could reflect sadness related to this loss of touch, confounding it with general unpleasantness. Including a specific question targeting “longing for touch” would have provided a more precise measure of participants’ emotional responses and their relationship to social touch. Future research should consider refining the scale or adding another scale to better capture distinct emotional dimensions, such as sadness and longing for touch, which may influence the perceived pleasantness of affective and non-affective touch. This refinement would enhance our understanding of the emotional impact of social touch, particularly in contexts like the COVID-19 pandemic, where its significance may be heightened.

Moreover, the current study did not employ a within-subject design. Thus, participants in the pre-COVID-19 group were not the same participants in the peri-COVID-19 group. For this reason, one cannot reach strong conclusions about causality, as other between-subject factors may have contributed to the between-group differences. However, both groups included first year psychology students from the University of Haifa and have the same inclusion criteria. Thus, one can assume that overall, both groups were similar, as between-subject differences are expected to balance out with random participant selection and assignment.

One limitation of the present study is the timing of participant recruitment during the pandemic. Due to government-imposed lockdowns and institutional restrictions during the early phase of COVID-19 (April–May 2020), we were unable to recruit participants at the initial outbreak. Consequently, our peri-COVID-19 data were collected between Israel’s second and third lockdowns. A more rigorous approach would have been to recruit participants at multiple peri-COVID-19 timepoints–an early phase (immediately following the outbreak) and a later phase–with sufficient sample sizes in each group. This would have allowed for a direct examination of the proposed temporal shift from hypervigilance to avoidance in attentional responses to social touch stimuli.

Overall, our results indicate that COVID-19 has modified how human beings perceive social touch. Our findings provide evidence for the impact of the pandemic on individuals’ perceptual and evaluative processes, highlighting the importance of considering social and environmental factors in understanding subjective experiences. Further research is needed to investigate the mechanisms underlying these effects and to determine the generalizability of these findings in different populations and contexts. Touch is vital for our social emotional development and provides essential tools for expressing our emotions, establishing intimacy and maintaining social bonds (Harlow and Zimmerman 1959). Therefore, studying and characterizing how the pandemic has altered social touch is crucial and can have a beneficial impact on how society functions as a whole.

## Data Availability

The datasets used and/or analysed during the current study are available from the corresponding author on reasonable request.
